# Decreased thymic output predicts progression of chronic kidney disease

**DOI:** 10.1186/s12979-023-00333-z

**Published:** 2023-02-14

**Authors:** Kenichiro Iio, Daijiro Kabata, Rei Iio, Shinichi Shibamoto, Yuuki Watanabe, Masashi Morita, Yosuke Imai, Masaki Hatanaka, Hiroki Omori, Yoshitaka Isaka

**Affiliations:** 1 Department of Nephrology, National Hospital Organization Osaka Minami Medical Center, 2-1 Kidohigashimachi Kawachinagano, Osaka, Japan; 2 Department of Medical Statistics, Osaka Metropolitan University Graduate School of Medicine, Osaka, Japan; 3 Department of Kidney Disease and Hypertension, Osaka General Medical Center, Osaka, Japan; 4 Department of Nephrology, Osaka University Graduate School of Medicine, Suita, Japan

**Keywords:** Chronic kidney disease, Recent thymic emigrants, T cell senescence, Renal outcomes

## Abstract

**Background:**

Chronic kidney disease (CKD) is age-related disease, and decreased renal function is associated with the premature aging of T cells and increased incidence of other age-related diseases. However, the relationship between T cell senescence and CKD progression remains unclear. Here, we investigated the relationship between T cell senescence, as indicated by decreased thymic output and increased proportion of highly differentiated CD28^−^ T cells, and CKD progression.

**Results:**

A total of 175 patients with non-dialysis-dependent CKD were enrolled in this study. Thymic output was assessed based on the CD45RA^+^CD31^+^CD4^+^ cell (recent thymic emigrant [RTE]) counts (RTEs) (/mm^3^) and the proportion of RTE among CD4^+^ T cells (RTE%). Highly differentiated T cells were assessed based on the proportion of CD28^−^ cells among CD4^+^ T cells (CD28^−^/CD4^+^) and CD28^−^ cells among CD8^+^ T cells (CD28^−^/CD8^+^). The primary outcome was estimated glomerular filtration rate (eGFR) decline of ≥40% or initiation of renal replacement therapy. The association between T cell senescence and renal outcomes was examined using Cox proportional hazards models and restricted cubic splines. The median age was 73 years, 33% were women, and the median eGFR was 26 mL/min/1.73 m^2^. The median RTEs, RTE%, CD28^−^/CD4^+^, and CD28^−^/CD8^+^ were 97.5/mm^3^, 16.2, 5.3, and 49.7%, respectively. After a median follow-up of 1.78 years, renal outcomes were observed in 71 patients. After adjusting for age, sex, eGFR, proteinuria, diabetes, and cytomegalovirus seropositivity, decreased RTEs, which corresponded to decreased thymic output, significantly and monotonically increased the risk of poor renal outcome (*p* = 0.04), and decreased RTE% and increased highly differentiated CD28^−^/CD4^+^ T cells also tended to monotonically increase the risk (*p* = 0.074 and *p* = 0.056, respectively), but not CD28^−^/CD8^+^ T cells.

**Conclusions:**

Decreased thymic output in CKD patients, as well as increased highly differentiated CD4^+^ T cells, predicted renal outcomes. Thus, the identification of patients prone to CKD progression using T cell senescence, particularly decreased RTE as a biomarker, may help to prevent progression to end-stage kidney disease.

**Supplementary Information:**

The online version contains supplementary material available at 10.1186/s12979-023-00333-z.

## Background

Chronic kidney disease (CKD), an age-related disease, affects 8–16% of the global population [1–3]. Age-related CKD progression is closely linked to senescence [2]. With aging, the kidney undergoes atrophy, glomerular sclerosis, and tubulointerstitial fibrosis [4]. The kidney of patients with CKD exhibits a high number of senescent cells [5], the accumulation of which disrupts renal function; therefore, the removal of these senescent cells has been shown to suppress glomerulosclerosis and preserve renal function in genetically engineered mouse models [6]. In addition, CKD progression induces the development of premature aging phenotypes, which include atherosclerosis, skeletal muscle mass loss, muscle wasting, and cognitive dysfunction [7–9]. In other words, CKD is not only an age-related disease but also accelerates aging.

Patients with CKD show lower thymic output and a higher proportion of T cells with advanced differentiation than individuals with normal renal function, which suggests advanced immune senescence [10–16]. Immune aging has been implicated in the pathogenesis of age-related diseases, including CKD [17–19]. In older individuals, the thymic output decreases, that is, recent thymic emigrants (RTE) decreases, thereby decreasing the number of naive T cells and increasing the number of memory subsets [20, 21]. The number of naive T cells is reduced less than expected because their numbers are maintained by homeostatic proliferation, but the diversity of T cell receptor (TCR) is reduced [20]. Memory T cells acquire a differentiated phenotype after repeated antigen stimulation [20, 22]. Age-related changes in T cells promote the accumulation of senescent cells, which leads to tissue injury owing to the insufficient clearance of senescent cells and induction of senescence-associated secretory phenotype (SASP) in senescent T cells [18, 20, 23–25]. However, the relationship between T cell senescence and CKD progression remains unclear, although immune senescence has been suggested to promote systemic and kidney aging and decline in renal function in mice [17, 18, 23, 24].

RTE are naive T cells that have recently emerged from the thymus and reflect its output [26]. During aging, the number of RTE decreases owing to thymic atrophy [25, 27]. In the elderly, the proportion of CD28^−^ T cells increases as a result of progressive differentiation of memory T cells due to lifelong antigen stimulation. Due to their inflammatory and cytotoxic characteristics, CD28^−^ T cells may cause tissue damage [20, 28]. Therefore, it is possible to evaluate the association of T cell senescence with CKD progression using RTE and CD28^−^ T cells as markers.

In this study, we investigated the relationship between T cell senescence, indicated by decreased RTE and increased CD28^−^ T cell proportion, and renal prognosis and mortality.

## Results

### T cell senescence and clinical characteristics

The distribution of the absolute number of RTE (RTEs)/mm^3^ and the proportion (%) of CD45RA^+^CD31^+^ cells among CD4^+^ cells (RTE%), CD28^−^ cells in the CD4^+^ cell population (CD28^−^/CD4^+^), and CD28^−^ cells in the CD8^+^ cell population (CD28^−^/CD8^+^) in 175 patients with a median follow-up period of 1.78 years are shown in Table 1.

**Table 1:** Recent thymic emigrants and CD28^−^ T cell parameters

Variable	Minimum	25%	50%	Mean	75%	Maximum	Missing (%)
RTEs	7.5	49.7	97.5	123.3	148.4	537.6	0
RTE%	1.8	9.7	16.2	18.0	24.2	53.7	0
CD28^−^/CD4^+^	0	1.7	5.3	9.1	12.0	56.0	15
CD28^−^/CD8^+^	3.3	29.6	49.7	48.4	66.6	88.6	1

*RTEs* recent thymic emigrants count per mm^3^, RTE% percentage of RTEs, *CD28*^*−*^*/CD4*^*+*^ percentage of CD28^−^ among CD4^+^ T cells, *CD28*^*−*^*/CD8*^*+*^ percentage of CD28^−^ among CD8^+^ T cells

Table 2 shows the baseline characteristics of the participants in this cohort and the divided groups of High and Low by median values of RTEs, RTE%, CD28^−^/CD4^+^, or CD28^−^/CD8^+^. Among all participants, the median age was 73 years (interquartile range, 62–79), 33% were female, the median estimated glomerular filtration rate (eGFR) was 26 (interquartile range, 16–40) mL/min/1.73 m^2^, and 94% were cytomegalovirus (CMV) seropositive. The Low RTEs and Low RTE% groups comprised older individuals, exhibited lower eGFR, and had higher proportions of males, higher number of patients with diabetes, and higher number of CMV seropositive. In contrast, there was little difference between the Low and High groups in terms of CD28^−^/CD4^+^ and CD28^−^/CD8^+^, while the Low group was all CMV seropositive. Since the presence of CMV is known to increase CD28^−^ T cells, the effect of CMV seropositivity on RTEs, RTE%, CD28^−^/CD4^+^, or CD28^−^/CD8^+^ was examined using multivariate regression analysis adjusted for age, sex, and eGFR. CD28^−^/CD8^+^ T cells was significantly more prevalent in CMV seropositive patients (β = 21.2, *p* = 0.004), while CD28^−^/CD4^+^ T cells tended to be more prevalent (β = 7.8, *p* = 0.10), although RTEs and RTE% were not associated with CMV seropositivity (β = 2.1, *p* = 0.95 and β = −2.4, *p* = 0.43, respectively) (Supplemental Table 1).

**Table 2:** Characteristics of the study population stratified by median RTEs, RTE%, CD28^−^/CD4^+^, and CD28^−^/CD4^+^

Variable	All	RTEs		RTE%		CD28^−^/CD4^+^		CD28^−^/CD8^+^	
		Low	High	Low	High	Low	High	Low	High
Range		7.5–97.5	97.6–537.6	1.8–16.2	16.3–53.7	0–5.3	5.4–56.0	3.3–49.7	49.8–88.6
N	175	88	87	88	87	75	74	87	86
Age (years)	73 [62, 79]	77 [71, 82]	68 [53, 76]	76 [71, 82]	68 [57, 76]	73 [63, 79]	76 [69, 82]	72 [55, 79]	75 [68, 81]
Sex (female), n (%)	58 (33)	21 (24)	37 (43)	22 (25)	36 (41)	16 (21)	26 (35)	26 (30)	31 (36)
Diabetes, n (%)	80 (46)	50 (57)	30 (34)	55 (63)	25 (29)	41 (55)	29 (39)	41 (47)	38 (44)
Cardiovascular disease, n (%)	43 (25)	31 (35)	12 (14)	29 (33)	14 (16)	21 (28)	20 (27)	20 (23)	23 (27)
Hypertension, n (%)	154 (88)	79 (90)	75 (86)	79 (90)	75 (86)	72 (96)	66 (89)	75 (86)	78 (91)
BMI (kg/m^2^)	23.9 [21.1, 25.8]	23.8 [21.4, 25.5]	24.1 [21.0, 26.3]	24.0 [21.8, 26.1]	23.4 [20.8, 25.6]	23.9 [22.1, 25.5]	24.3 [22.1, 26.4]	23.9 [20.9, 26.1]	23.9 [21.8, 25.5]
Chronic glomerulonephritis, n (%)	47 (27)	16 (18)	31 (36)	17 (19)	30 (34)	13 (17)	18 (24)	27 (31)	18 (21)
Diabetic nephropathy, n (%)	54 (31)	35 (40)	19 (22)	36 (41)	18 (21)	27 (36)	21 (28)	26 (30)	28 (33)
Nephrosclerosis, n (%)	45 (26)	28 (32)	17 (20)	28 (32)	17 (20)	24 (32)	20 (27)	20 (23)	25 (29)
Others, n (%)	29 (17)	9 (10)	20 (23)	7 (8)	22 (25)	11 (15)	15 (20)	14 (16)	15 (17)
Current smoking, n (%)	27 (16)	12 (14)	15 (17)	15 (18)	12 (14)	14 (19)	10 (14)	15 (18)	12 (14)
Use of ACE-inhibitor or ARB, n (%)	100 (57)	49 (56)	51 (59)	52 (59)	48 (55)	43 (57)	42 (57)	48 (55)	51 (59)
CMV seropositive, n (%)	164 (94)	85 (97)	79 (92)	85 (97)	79 (92)	70 (93)	73 (100)	78 (90)	85 (100)
eGFR (mL/min/1.73 m^2^)	26 [16, 40]	22 [13, 32]	32 [22, 48]	23 [14, 33]	31 [17, 47]	26 [15, 37]	26 [18, 36]	29 [15 42	26 [16, 34]
CKD stage 1 or 2, n (%)	14 (8)	3 (3)	11 (13)	2 (2)	12 (14)	3 (4)	3 (4)	10 (11)	3 (3)
CKD stage 3, n (%)	61 (35)	23 (26)	38 (44)	26 (30)	35 (40)	27 (36)	25 (34)	32 (37)	28 (33)
CKD stage 4, n (%)	60 (34)	34 (39)	26 (30)	36 (41)	24 (28)	26 (35)	32 (43)	26 (30)	34 (40)
CKD stage 5, n (%)	40 (23)	28 (32)	12 (14)	24 (27)	16 (18)	19 (25)	14 (19)	19 (22)	21 (24)
Urinary protein (g/gCr)	1.25 [0.40, 2.83]	1.60 [0.60, 3.41]	0.82 [0.24, 2.67]	1.72 [0.55, 3.60]	0.83 [0.28, 1.96]	1.25 [0.32, 3.03]	1.23 [0.49, 2.66]	1.25 [0.34, 3.24]	1.26 [0.48, 2.68]
Hemoglobin (g/dL)	12.1 [10.7, 13.7]	11.2 [10.5, 13.2]	12.6 [11.4, 14.0]	11.5 [10.5, 13.4]	12.4 [10.9, 14.0]	11.7 [10.6, 13.7]	12.4 [11.0, 14.0]	12.1 [10.7, 13.6]	12.1 [10.6, 13.9]
Serum albumin (g/dL)	4.0 [3.6, 4.2]	3.9 [3.5, 4.1]	4.1 [3.6, 4.3]	3.9 [3.4, 4.2]	4.0 [3.6, 4.3]	4.0 [3.7, 4.2]	4.0 [3.7, 4.3]	4.0 [3.5, 4.3]	3.9 [3.6, 4.2]
Serum C-reactive protein (mg/dL)	0.07 [0.04, 0.13]	0.08 [0.03, 0.12]	0.07 [0.04, 0.14]	0.07 [0.04, 0.15]	0.07 [0.04, 0.15]	0.07 [0.04, 0.11]	0.07 [0.03, 0.13]	0.07 [0.04, 0.11]	0.08 [0.03, 0.15]

Data are presented as numbers (%) or median [interquartile range]

*BMI* body mass index, *ACE* angiotensin-converting enzyme, *ARB* angiotensin receptor blockers, *CMV* cytomegalovirus, *eGFR* estimated glomerular filtration rate, *CKD* chronic kidney disease

### Renal outcomes

**Fig. 1:**
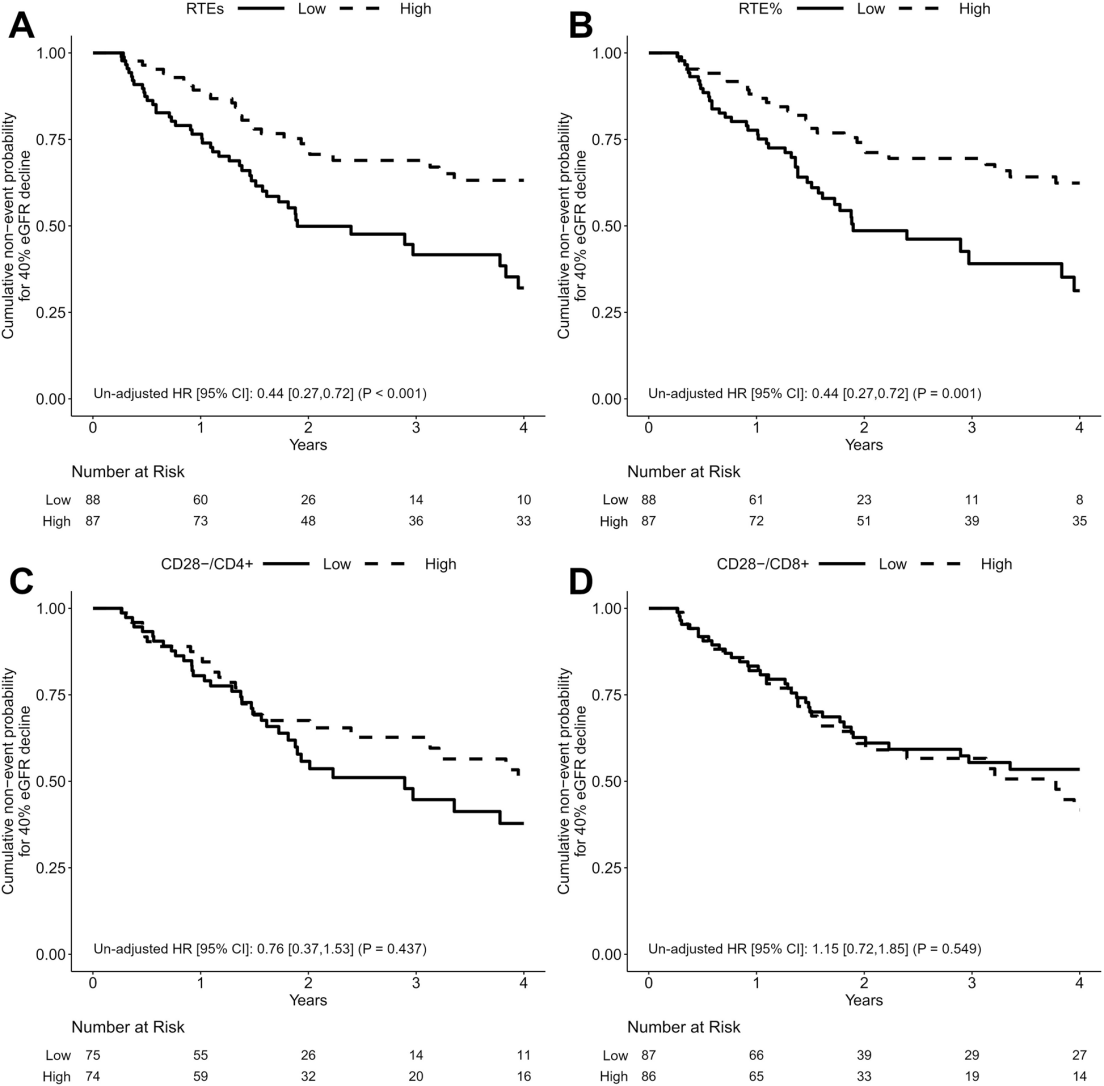
Kaplan-Meier survival curves for 40% eGFR decline divided based on median values of (**A**) recent thymic emigrant count, (**B**) proportion of recent thymic emigrants among CD4^+^ T cells, (**C**) proportion of CD28^−^ cells among CD4^+^ T cells, and (**D**) proportion of CD28^−^ cells among CD8^+^ T cells. High recent thymic emigrant count (RTEs) groups and High proportion of RTEs among CD4^+^ cells (RTE%) groups were significantly associated with a lower risk of ≥40% eGFR decline. RTEs, RTE%, the proportion of CD28^−^ cells among CD4^+^ T cells (CD28^−^/CD4^+^), and the proportion of CD28^−^ cells among CD8^+^ T cells (CD28^−^/CD8^+^) were divided into High and Low groups based on median values. Unadjusted hazard ratios (HRs) were calculated using Cox proportional hazards regression models

**Fig. 2:**
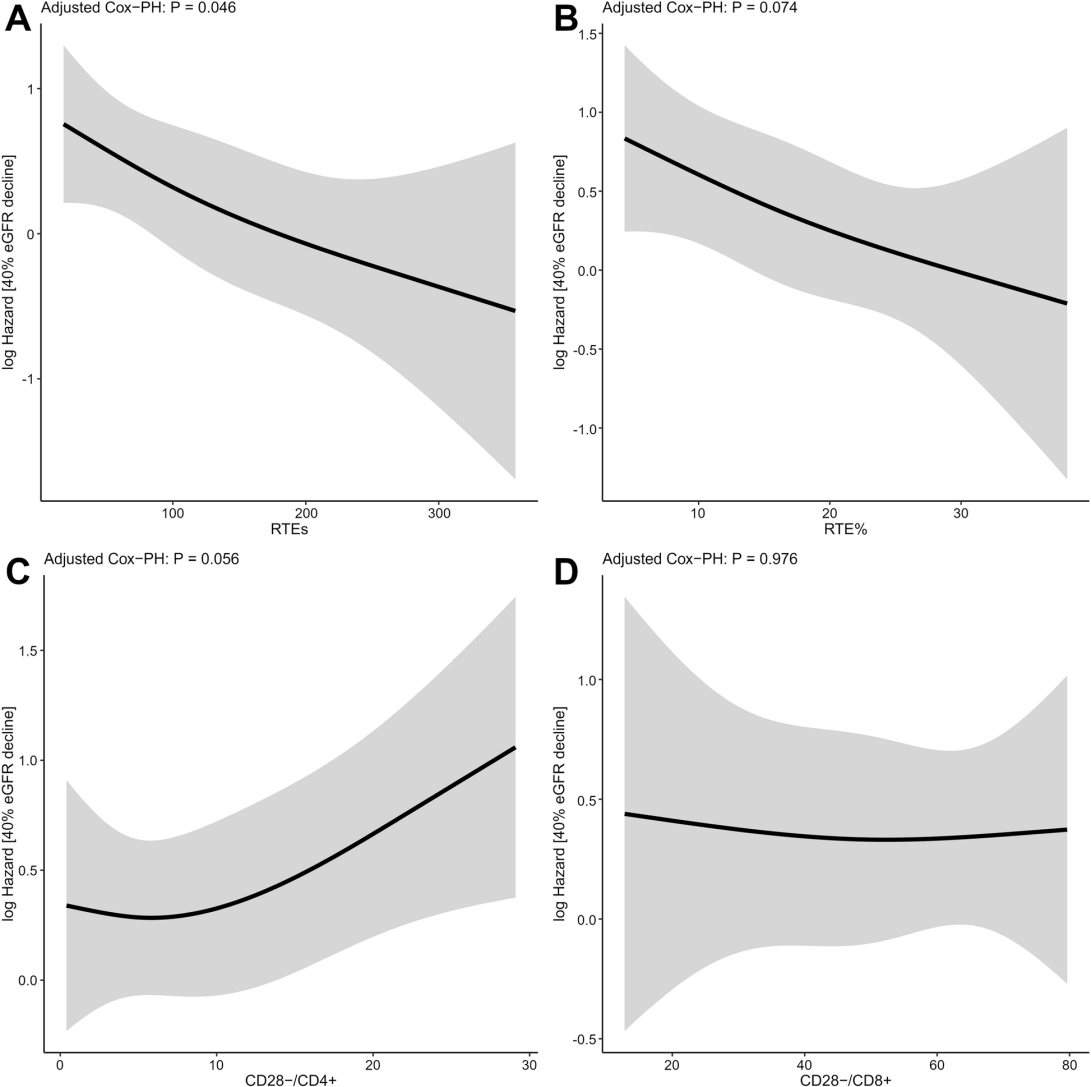
Association of risk of ≥40% eGFR decline with (**A**) recent thymic emigrants count, (**B**) proportion of recent thymic emigrants among CD4^+^ T cells, (**C**) proportion of CD28^−^ cells among CD4^+^ T cells, and (**D**) proportion of CD28^−^ cells among CD8^+^ T cells. Models were performed using Cox proportional hazards regression models with restricted cubic splines. A linear association was observed between recent thymic emigrants count (RTEs), proportion of RTEs among CD4^+^ T cells (RTE%), and proportion of CD28^−^ cells among CD4^+^ T cells (CD28^−^/CD4^+^) with the risk of CKD progression. Decreased RTEs were associated with a higher risk of CKD progression (*p* = 0.04). Decreased RTE% and increased CD28^−^/CD4^+^ T cells tended to be associated with CKD progression. Models were adjusted for age, sex, estimated glomerular filtration rate (GFR) urinary protein, diabetes, and cytomegalovirus seropositive. The solid line indicates the log hazard ratio, and the grey area indicates 95% confidence interval

**Fig. 3:**
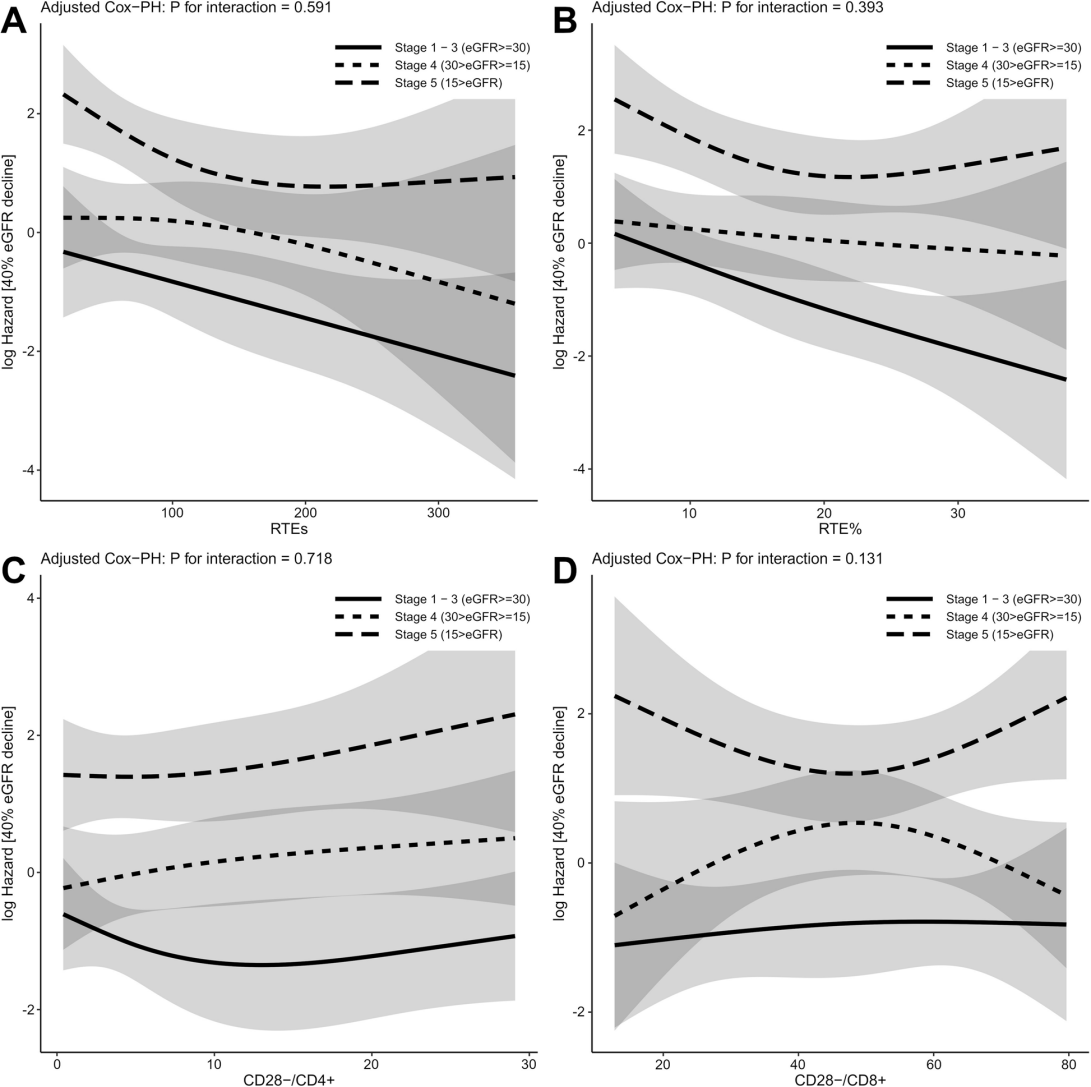
Association of risk of ≥40% eGFR decline with (**A**) recent thymic emigrants count, (**B**) proportion of recent thymic emigrants among CD4^+^ T cells, (**C**) proportion of CD28^−^ cells among CD4^+^ T cells, and (**D**) proportion of CD28^−^ cells among CD8^+^ T cells separately compared for CKD stages 1–3, 4, and 5. Models were constructed using Cox proportional hazards regression models with restricted cubic splines. There was no significant interaction in the risk of ≥40% eGFR decline for recent thymic emigrant count (RTEs), proportion of recent thymic emigrants among CD4^+^ T cells (RTE%), proportion of CD28^−^ cells among CD4^+^ T cells (CD28^−^/CD4^+^), and proportion of CD28^−^ cells among CD8^+^ T cells (CD28^−^/CD8^+^), irrespective of chronic kidney disease (CKD) stages 1–3, 4, and 5. Models were adjusted for age, sex, estimated glomerular filtration rate (eGFR) urinary protein, diabetes, and cytomegalovirus seropositive. Solid and dotted lines indicate log hazard ratios, and gray areas indicate 95% confidence intervals

The primary outcome of a 40% reduction in eGFR or initiation of renal replacement therapy, occurred in 71 of 175 patients. Survival curves were generated for the Low and High groups divided by the median values of RTEs, RTE%, CD28^−^/CD4^+^, and CD28^−^/CD8^+^ (Fig. 1). Patients in the High RTEs and the High RTE% groups had a lower risk of poor renal outcome as determined using univariate Cox proportional hazards analysis [hazard ratio (HR) 0.44 (95% confidence interval (CI): 0.27–0.72, *p* < 0.001) and HR 0.44 (95% CI: 0.27–0.72, *p* < 0.001), respectively]. In contrast, the effect of CD28^−^/CD4^+^ and CD28^−^/CD8^+^ on renal prognosis did not significantly differ between the High and Low groups. To demonstrate the relationship between T cell senescence and renal prognosis in CKD, we modeled RTEs, RTE%, CD28^−^/CD4^+^, and CD28^−^/CD8^+^ as continuous variables using a multivariate Cox proportional hazards model with restricted cubic splines (Fig. 2). Models were adjusted for age, sex, eGFR, urinary protein, diabetes, and CMV seropositive. The results showed a linear association between RTE, RTE%, and CD28^−^/CD4^+^ with the risk of CKD progression. Lower RTEs were associated with a significantly higher risk of CKD progression (*p* = 0.04), and lower RTE% tended to be associated (*p* = 0.074). Univariate analysis showed no association between CD28^−^/CD4^+^ T cells and CKD progression (Fig. 1), although elevated CD28^−^/CD4^+^ T cells tended to be associated with a higher risk in multivariate analysis (*p* = 0.056). In contrast, CD28^−^/CD8^+^ T cells were not significantly associated with CKD progression. We then examined whether CKD progression differed by CKD stages 1–3, 4, and 5 using Cox proportional hazards regression models with restricted cubic splines. Regardless of RTEs, RTE%, CD28^−^/CD4^+^, and CD28^−^/CD8^+^, no significant interaction was observed between CKD stages 1–3, 4, or 5 in CKD progression (Fig. 3). These results suggest that a decrease in thymic output was associated with the progression of CKD regardless of the CKD stage, whereas an increase in proportion of highly differentiated CD4^+^ T cells only tended to be associated, and an increase in the proportion of highly differentiated CD8^+^ T cells was not associated.

### All-cause mortality

**Fig. 4:**
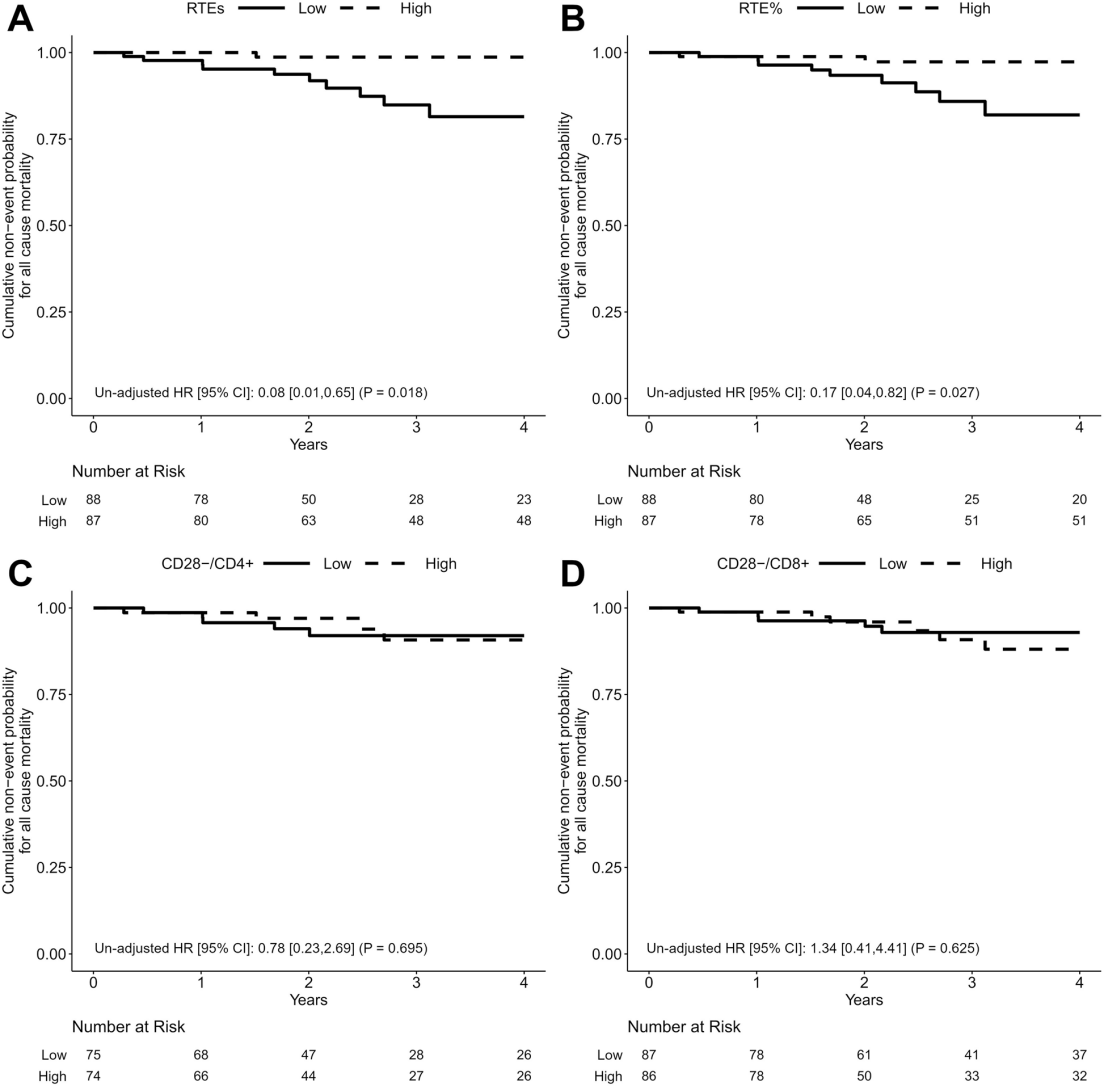
Kaplan-Meier survival curves for all-cause mortality divided based on median value of (**A**) recent thymic emigrant count, (**B**) proportion of recent thymic emigrants among CD4^+^ T cells, (**C**) proportion of CD28^−^ cells among CD4^+^ T cells, and (**D**) proportion of CD28^−^ cells among CD8^+^ T cells. High recent thymic emigrant count (RTEs) groups and High proportion of RTEs among CD4^+^ T cells (RTE%) groups were significantly associated with a lower risk of all-cause mortality. RTEs, RTE%, the proportion of CD28^−^ cells among CD4^+^ T cells (CD28^−^/CD4^+^), and the proportion of CD28^−^ cells among CD8^+^ T cells (CD28^−^/CD8^+^) were divided into High and Low groups based on median values. Unadjusted hazard ratios (HRs) were calculated using Cox proportional hazards regression models

During the follow-up period, all-cause deaths occurred in 11 patients. Figure 4 shows the cumulative survival curves stratified by median values of RTE, RTE%, CD28^−^/CD4^+^, and CD28^−^/CD8^+^. Univariate Cox proportional hazards analysis showed significantly decreased risk of all-cause deaths in the High RTE and High RTE% groups [HR 0.08 (95% CI: 0.01–0.65, *p* = 0.018) and HR 0.17 (95% CI: 0.04–0.82, *p* = 0.027), respectively]. All-cause mortality did not differ between the High and Low groups for CD28^−^/CD4^+^ and CD28^−^/CD8^+^. Therefore, decreased thymic output was associated with increased all-cause mortality. Multivariate analysis with all-cause mortality as the outcome was not performed because the number of events in the cohort was too small to estimate an appropriate regression model without overfitting.

## Discussion

In this study, we explored the impact of T cell senescence on the renal prognosis and mortality of patients with CKD. We found that decreased RTE, which corresponds to decreased thymic output, was associated with CKD progression and high mortality, and an increase in highly differentiated CD28^−^CD4^+^ T cells, which increases with age [20], tended to be associated with CKD progression. Even when non-linearities were considered in analyzing renal outcomes, decreased RTE and increased CD28^−^/CD4^+^ T cells were monotonically associated with CKD progression. However, highly differentiated CD28^−^CD8^+^ cells were not associated with CKD progression or high mortality. These results suggest that decreased thymic output in patients with CKD predicts worse renal outcomes and high mortality, whereas for highly differentiated T cells, the results only suggest that CD28^−^CD4^+^ T cells can predict CKD progression.

Thymic atrophy is a characteristic of an aging immune system and has been implicated in age-related diseases such as infection, malignancy, atherosclerosis, and CKD [13, 25, 29–32]. However, epidemiologic data are limited in patients with non-dialysis-dependent CKD [25]. To our knowledge, this is the first study to demonstrate the impact of decreased thymic output on renal prognosis and all-cause mortality in patients with non-dialysis-dependent CKD.

An immunological model has been constructed using epidemiological and immunological data to show that an age-related decrease in thymic output is associated with the development of infectious diseases and malignancies [33]. Decreased RTE in renal transplant patients increases all-cause mortality [30, 31]. In dialysis patients, decreased RTE is associated with death caused by infection [13], and decreased naive T cell count due to thymic atrophy increase all-cause mortality [34, 35]. Furthermore, our results are consistent with previous findings showing that shorter telomeres in peripheral blood leukocytes worsen renal prognosis and mortality, because the decrease in naive T cell counts due to thymic atrophy is compensated by increased T cell division, thereby shortening telomeres [36–39]. In the present study, the patients in the Low RTEs and Low RTE% groups had decreased thymic output, were older, had lower eGFR, and had a higher prevalence of diabetes and, thus, presented an advanced aging phenotype, consistent with previous findings [10, 13, 40].

There are three potential mechanisms by which decreased thymic output contributes to CKD progression and increased mortality. (a) Low thymic output reduces the diversity of TCR repertoire and may reduce the clearance of senescent cells by immune cells. CD8^+^ T cells eliminate senescent cells [24]; perforin-knockout mice with impaired cytotoxic activity show accumulation of senescent cells, chronic inflammation, and accelerated glomerular stiffening in the kidney [23]. Hepatocytes in a precancerous state are eliminated by monocytes and macrophages via CD4^+^ T cells [41], and senescent cells are also eliminated by the similar mechanism [42, 43]. In a previous study showing that the removal of p16^Ink4a^-positive cells prevented glomerulosclerosis, a renal aging phenotype, and attenuated the decline in renal function, SA-β-Gal-positive cells were present in tubular epithelial cells, suggesting that the removal of senescent cells in tubular epithelial cells may prevent renal aging [6]. (b) When the number of newly produced naive T cells decreases, homeostatic proliferation maintains the peripheral T cell count [20, 21]. However, homeostatic proliferation increases the percentage of CD153^+^PD1^+^CD4^+^ T cells, a dysfunctional memory-type T cell in mice, although this has not yet been reported in humans [22]. Since thymic atrophy decreases the number of newly produced naive T cells, the number of these senescence-associated T (SA-T) cells may therefore increase with thymic atrophy [22]. SA-T cells secrete inflammatory chemokines involved in chronic tissue inflammation, delay kidney tissue repair after acute kidney injury [44], and may be associated with CKD progression. (c) Thymic atrophy may reflect a systemic state of aging. As aging progresses, local and systemic inflammation induced by SASP causes organ damage, which may cause thymic atrophy and decreases kidney function, and increase mortality [45]. Conversely, the presence of CKD decreases thymic output [10], increasing the susceptibility to further progression of CKD and high mortality.

Senescence promotes T cell differentiation, which disrupts T cell homeostasis in peripheral tissues [21]. Therefore, an increase in highly differentiated CD28^−^ T cells may reflect age-related changes in peripheral tissues. In addition, CD28^−^ T cells have inflammatory and cytotoxic characteristics that may cause tissue damage [20]. However, in the present study, increased proportion of CD28^−^CD4^+^ T cells tended to be associated only with CKD progression, but CD28^−^CD8^+^ T cells did not. One possible reason is that the proportion of CD28^−^ T cells in tissues differs from that in circulating blood [46]. CD28^−^ T cells are abundant in tissues during age-associated inflammation [20], while the circulating blood contains T cells derived from inflammation in organs other than the kidney, and therefore may not accurately reflect kidney aging. Another possibility is that increase in T cells, such as SA-T cells in mice, due to decreased thymic output may have a greater impact on age-related disease onset and progression than increase in CD28^−^ T cells, although the corresponding subpopulation remains to be identified in humans [22]. Furthermore, in a previous study examining mortality after kidney transplantation, decreased RTE significantly predicted mortality, while an increased CD28^−^ T cells showed a trend, but was not significant [31], suggesting that RTE is a better predictor than CD28^−^ T cells. This study has several limitations. First, a causal relationship between decreased thymic outcome and CKD progression could not be established as this was an observational study. Second, this was a single-center study with a limited number of patients, and the results may have lacked statistical power to confirm a relationship between CD28^−^ T cells and poor renal outcomes or mortality. Mortality was investigated using a univariate analysis because the number of events in the cohort was insufficient to estimate a suitable regression model without overfitting. Third, although the multivariate analysis was adjusted for possible confounders, associations with other factors cannot be excluded.

## Conclusions

In patients with CKD, decreased RTE was associated with CKD progression and high mortality. Therefore, it may be possible to prevent progression to end-stage renal disease using decreased RTE as a biomarker to identify patients prone to CKD progression and to provide early multidisciplinary treatment. Additionally, thymic atrophy caused by CKD may partly account for the high frequency of cardiovascular complications, infections, and progressive frailty in patients with CKD. Therefore, targeting thymic atrophy is a potential strategy to control CKD progression and associated complications.

## Methods

### Study population

This prospective observational study was conducted at the Osaka Minami Medical Center, Kawachinagano, Japan. Details of the cohort have been described previously [12]. Briefly, between June 2013 and August 2021, we enrolled 175 patients with non-dialysis-dependent CKD aged ≥20 years. The sample size was determined to obtain a sufficient number of outcomes for multivariate analysis. The patients were followed up for 48 months. To avoid the influence of acute declines in renal function and inflammation, we excluded patients with acute kidney injury, those on immunosuppressive drugs, those with active infection, or those with coexisting inflammatory diseases such as systemic lupus erythematosus, rheumatoid arthritis, and vasculitis. We also excluded patients who were followed up for <3 months.

### Data collection

Baseline characteristics at the initiation of the study were collected from the medical records of our hospital, which included age, sex, body mass index [BMI; weight (kg)/height (m^2^)], cause of CKD (chronic glomerulonephritis, diabetic nephropathy, nephrosclerosis, or other), smoking status, use of angiotensin-converting enzyme inhibitors or angiotensin II receptor blockers, eGFR, urinary protein, hemoglobin, serum levels of albumin and C-reactive protein, presence or absence of hypertension and diabetes, and history of cardiovascular disease. eGFR (mL/min/1.73 m^2^) was calculated using the following equation [47]: 194 × age (years)^−0.287^ × serum creatinine (mg/dL)^−1.094^ (×0.739 if female). A history of stroke, coronary artery disease, or peripheral arterial disease was defined as a history of cardiovascular disease.

### Clinical outcomes

The primary outcome was ≥40% decline in eGFR or the initiation of renal replacement therapy. The secondary outcome was all-cause mortality.

### T cell phenotyping

T cells in peripheral blood mononuclear cells (PBMCs) or peripheral blood was detected using flow cytometry. PBMCs were separated using Ficoll-Paque PLUS (GE Healthcare, Uppsala, Sweden) and stored in liquid nitrogen. After thawing, PBMCs were washed twice in RPMI (Wako Pure Chemical Industries, Osaka, Japan) containing 10% fetal calf serum (complete medium). Peripheral blood and PBMCs were stained with the following conjugated antibodies: anti-human CD3-PE-CyA7 (mouse IgG1 k, clone UCHT1, Biolegend, Ozyme, Saint-Quentin–en-Yvelines, France, #300420), CD4-FITC (mouse IgG1 k, clone RPA-T4, Biolegend, #300506), CD8-PerCP-Cy5.5 (mouse IgG1 k, clone RPA-T8, Biolegend, #301032), CD28-APC (mouse IgG1 k, clone CD28.21, Biolegend, #302911), CD31-PE (mouse IgG1 k, clone WM59, Biolegend, #303106), and CD45RA-ECD (mouse IgG1, clone 2H4LDH11LDB9, Beckman-Coulter, Villepinte, France, #IM2711U). Flow cytometric analysis was performed using Navios (Beckman), and data were analyzed using the FlowJo software (Treestar, San Carlos, CA, USA). CD45RA^+^CD31^+^CD4^+^ T cells were defined as RTE, as previously described [26, 27]. The absolute number of RTE was expressed as RTEs/mm^3^, and the proportion of CD45RA^+^CD31^+^ cells among CD4^+^ T cells was expressed as RTE%. The number of RTEs was calculated from the peripheral blood lymphocyte count at the time of sample collection. The proportion of CD28^−^ cells in the CD4^+^ T cell population was expressed as CD28^−^/CD4^+^ (%) and that in the CD8^+^ T cell population was expressed as CD28^−^/CD8^+^ (%). The gating strategy for RTE, CD28^−^CD4^+^, and CD28^−^CD8^+^ T cells is shown in Supplemental Fig. 1.

### Statistical analysis

We summarized the baseline distributions of RTEs, RTE%, CD28^−^/CD4^+^, and CD28^−^/CD8^+^ using mean, minimum, maximum, and quartile points. Patient demographics and baseline clinical characteristics were summarized using median and interquartile range for continuous variables, and number and proportion (%) are used for categorical variables.

We estimated the cumulative event-free probability of ≥40% eGFR reduction based on the product limit estimation method. Patients were divided into High and Low groups using the median value of RTEs, RTE%, CD28^−^/CD4^+^, or CD28^−^/CD8^+^, and the Cox proportional hazards regression model was employed to compare the survival curves between the Low and High groups. Furthermore, we evaluated the non-linear effects of RTEs and RTE% on the occurrence of ≥40% eGFR reduction using a multivariable Cox proportional hazard regression model by adjusting for age, sex, eGFR, urinary protein, diabetes separately, and CMV seropositivity at the baseline. In addition, the effects of CD28^−^/CD4^+^ and CD28^−^/CD8^+^ on the primary outcome were assessed using similar regression models. To determine whether the relationships differed based on the baseline CKD stage, we performed similar regression analyses, considering a cross-product term between the main exposure and the CKD stage group. We treated patients with CKD stages 1–3 as a single group because of limited sample size. The non-linear effects were estimated using a restricted cubic spline method with three knots. Patients were censored at death or at the end of the follow-up period.

Additionally, we assessed the cumulative survival probability using the product limit estimation method and compared the difference in the survival curves between the subject groups using the unadjusted Cox proportional hazards regression model. Although we attempted to estimate the adjusted effect of the number of cells on the all-cause mortality, we could not estimate the appropriate regression model without overfitting because there were few events in our cohort.

To impute missing values in the above regression models, we employed a multiple imputation approach based on the predictive mean matching method with five repetitions. All statistical tests were conducted with a two-sided 5% significance level using R software (https://cran.r-project.org/).

## Supplementary Information

**Additional file 1: Fig. S1.** Gating strategy for recent thymic emigrants (RTE) and CD28^−^CD4^+^ and CD28^−^CD8^+^ cells. **Table S1.** Association between the number of recent thymic emigrants (RTEs), the proportion of RTEs among CD4^+^ T cells (RTE%), the proportion of CD28^−^ cells among CD4^+^ T cells (CD28^−^/CD4^+^), the proportion of CD28^−^ cells among CD4^+^ T cells (CD28^−^/CD8^+^) with cytomegalovirus (CMV) seropositivity.

## Data Availability

All data generated or analyzed during this study are included in this published article.

## Abbreviations

CKD: Chronic kidney disease
SASP: Senescence-associated secretory phenotype
TCR: T cell receptor
RTE: Recent thymic emigrants
RTEs: The absolute number of RTE
RTE%: The proportion of CD45RA^+^CD31^+^ cells among CD4^+^ T cells
CD28^−^/CD4^+^: The proportion of CD28^−^ cells in the CD4^+^ T cell population
CD28^−^/CD8^+^: The proportion of CD28^−^ cells in the CD8^+^ T cell population
eGFR: Estimated glomerular filtration rate
CMV: Cytomegalovirus
CI: Confidence interval
SA-T: Senescence-associated T
PBMCs: Peripheral blood mononuclear cells
