# Supplementary material for: Decreased thymic output predicts progression of chronic kidney disease
Source: Immun Ageing. 2023 Feb 14;20:8. doi: 10.1186/s12979-023-00333-z (PMC9926722; doi:10.1186/s12979-023-00333-z)
Supplement: Supplementary file 1 — Additional file 1: Fig. S1. Gating strategy for recent thymic emigrants (RTE) and CD28−CD4+ and CD28−CD8+ cells. Table S1. Association between the number of recent thymic emigrants (RTEs), the proportion of RTEs among CD4+ T cells (RTE%), the proportion of CD28− cells among CD4+ T cells (CD28−/CD4+), the proportion of CD28− cells among CD4+ T cells (CD28−/CD8+) with cytomegalovirus (CMV) seropositivity. [file 12979_2023_333_MOESM1_ESM.docx]

**Supplementary Information**

**Decreased thymic output predicts progression of chronic kidney disease**

Kenichiro Iio^1^, Daijiro Kabata^2^, Rei Iio^3^, Shinichi Shibamoto^1^, Yuuki Watanabe^1^, Masashi Morita^1^, Yosuke Imai^1^, Masaki Hatanaka^1^, Hiroki Omori^1^, Yoshitaka Isaka^4^

^1^Department of Nephrology, National Hospital Organization Osaka Minami Medical Center, Kawachinagano, Japan

^2^Department of Medical Statistics, Osaka Metropolitan University Graduate School of Medicine, Osaka, Japan

^3^Department of Kidney Disease and Hypertension, Osaka General Medical Center, Osaka, Japan

^4^Department of Nephrology, Osaka University Graduate School of Medicine, Suita, Japan

**Corresponding Author:**

Kenichiro Iio

Department of Nephrology, National Hospital Organization Osaka Minami Medical Center, 2-1 Kidohigashimachi Kawachinagano Osaka 586-8521, Japan.

Phone: +81-721-53-5761

Fax: +81-721-53-8904

E-mail: [iioken16@outlook.com](mailto:iioken16@outlook.com)

**Supplemental Figure 1.** Gating strategy for recent thymic emigrants (RTE) and CD28^-^CD4^+^ and CD28^-^CD8^+^ T cells.

**Supplemental Table 1.** Association between the number of recent thymic emigrants (RTEs), the proportion of RTEs among CD4^+^ T cells (RTE%), the proportion of CD28^-^ cells among CD4^+^ T cells (CD28^-^/CD4^+^), the proportion of CD28^-^ cells among CD4^+^ T cells (CD28^-^/CD8^+^) with cytomegalovirus (CMV) seropositive.

|  | β | 95% confidence interval |  | P |
| --- | --- | --- | --- | --- |
| RTEs | 2.1 | -62.0 to 66.2 |  | 0.95 |
| RTE% | -2.4 | -8.5 to 3.6 |  | 0.43 |
| CD28^-^/CD4^+^ | 7.8 | -1.6 to 17.2 |  | 0.10 |
| CD28^-^/CD8^+^ | 21.2 | 7.1 to 35.3 |  | 0.004 |

Multivariate regression analysis was adjusted for age, sex, and eGFR.

eGFR, estimated glomerular filtration rate.
